# War at Sea: Burn Care Challenges—Past, Present and Future

**DOI:** 10.3390/ebj4040041

**Published:** 2023-12-11

**Authors:** Matthew D. Tadlock, Theodore D. Edson, Jill M. Cancio, Dana M. Flieger, Aaron S. Wickard, Bailey Grimsley, Corey G. Gustafson, Jay A. Yelon, James C. Jeng, Jennifer M. Gurney

**Affiliations:** 1 Department of Surgery, Naval Medical Center, San Diego, CA 92134, USA; aaronwickard@gmail.com (A.S.W.); baileymgrimsley@gmail.com (B.G.); 2 1st Medical Battalion, 1st Marine Logistics Group, Camp Pendleton, CA 92058, USA; theodore.d.edson@gmail.com; 3 U.S. Army Burn Center, U.S. Army Institute of Surgical Research, Ft. Sam Houston, San Antonio, TX 78234, USA; jill.m.cancio.civ@health.mil; 4 Navy Medicine Readiness and Training Command, Camp Lejeune, NC 28547, USA; dfliegs@gmail.com; 5 Navy Personnel Command, Millington, TN 38054, USA; corey.g.gustafson.mil@us.navy.mil; 6 Navy Medical Operational Command, University of Pennsylvania, Philadelphia, PA 19104, USA; jayelon@gmail.com; 7 Division of Trauma Critical Care, Acute Care & Burn Surgery, Department of Surgery, University of California Irvine, Orange, CA 92868, USA; jcjeng@hs.uci.edu; 8 Joint Trauma System, DoD Center of Excellence for Trauma, Joint Base San Antonio-Fort Sam, Houston, TX 78234, USA; jennifer.m.gurney.mil@health.mil

**Keywords:** burns, mass-casualty disaster, maritime injury, armed conflict, distributed maritime operations, austere, critical care, prolonged casualty care, freeze-dried plasma, whole blood

## Abstract

Throughout history, seafarers have been exposed to potential thermal injuries during naval warfare; however, injury prevention, including advances in personal protective equipment, has saved lives. Thankfully, burn injuries have decreased over time, which has resulted in a significant clinical skills gap. Ships with only Role 1 (no surgical capability) assets have worse outcomes after burn injury compared to those with Role 2 (surgical capability) assets. To prepare for future burn care challenges during a war at sea, Military Medicine must re-learn the lessons of World War I and World War II. Burn injuries do not occur in isolation during war and are associated with concomitant traumatic injuries. To care for burn casualties at sea, there is an urgent need to increase the availability of whole blood and dried plasma, resuscitation fluids that were ubiquitous throughout the naval force during World War II for both hemorrhagic and burn shock resuscitation. Furthermore, those providing trauma care at sea require formal burn care training and skills sustainment experiences in the clinical management of Burn, Trauma, and Critical Care patients. While burn education, training, and experience must be improved, modern high-energy weapons systems and anti-ship ballistic missiles necessitate concurrent investments in prevention, countermeasures, and personal protective equipment to decrease the likelihood of burn injury and damage resulting from these attacks.

## 1. Introduction

While serious burn-related injuries are an infrequent occurrence during routine naval operations, the risk of thermal injury from high-pressure steam, electrical injuries, flash burns, toxic gas inhalation, smoke inhalation, chemical-related burns, jet-blast, and explosions remains ever-present for those going to sea [1,2,3,4,5,6,7]. Since the first seafarers began navigating the world’s oceans, the naval warship has been an instrument of diplomacy and war, either transporting combatants or engaging in direct action on the high seas [8,9,10,11,12,13]. As the warship evolved, it has represented a paradox to the individual sailor, protecting them from the perils of the ocean, severe weather, and combat but, once aflame, it becomes a deadly environment where the inhospitable sea may be the sailor’s only refuge. While medical care, especially burn care, is not the primary purpose of the warship, it becomes a priority once a casualty-producing event occurs.

The U.S. Navy has not been engaged in large-scale combat operations at sea since World War II. Ground-based conflicts have produced multiple lessons learned that have been inculcated into military and civilian trauma systems. In the eponymous ‘Walker Dip’, the former Surgeon General of the British Armed Forces describes how and why the life-saving lessons learned in combat casualty care are lost during interwar periods and subsequently need to be relearned by new generations of Military Medicine caregivers [14]. These lessons learned in burn care, earned in blood and treasure during war at sea, have been experiencing a Walker Dip since 1945. The purpose of this review is to learn from the history of thermal injury at sea and provide a broad overview of maritime-related burn injury management to prepare the next generation of shipboard caregivers.

## 2. Naval Warfare, Medical Tactics and Burn Care at Sea: First and Second World Wars

### 2.1. World War I

During naval action in the Age of Sail, wounded were typically cared for by the Ship’s Surgeon below the waterline, safe from enemy fire on the orlop deck (the lowest deck on a multi-deck sailing ship), in the cockpit or junior officer’s quarters. Prior to World War I (WWI), as steam replaced sails and the engine room replaced the cockpit, shipboard medical care during naval action occurred in wardrooms above the waterline. As shipboard medical providers were often killed or injured and medical supplies were often destroyed during casualty care, the navies of the world began incorporating dedicated casualty care spaces, called Battle Dressing Stations (BDS), below the waterline. By 1908, the requirement for at least two BDS became formal policy in the U.S. Navy and they were incorporated on all warships; currently, all U.S. warships have BDS, the number of which is based on standard ship crew complements [8,15].

During the 1916 Battle of Jutland, the paradox of the naval warship as a protector from the sea that could also become a fiery death trap became readily apparent when German shells ignited improperly stowed cordite in the gun turrets of three lightly armored British battle cruisers. Fires spread rapidly down to the magazines, causing explosions resulting in immediate death for some and severe burns or toxic gas inhalation for the survivors. Over 6000 British sailors, many of whom drowned, perished at Jutland [6,15,16]. This massive loss of human life was not unusual during the wars of the 20th century. However, when examining this scale of human carnage through a 21st century lens, particularly in the context of technologically enabled instantaneous information exchange, this type of tragedy could impact perceptions of acceptable risk tolerance and sap national will and its commitment to wartime activities. 

**Figure 1:**
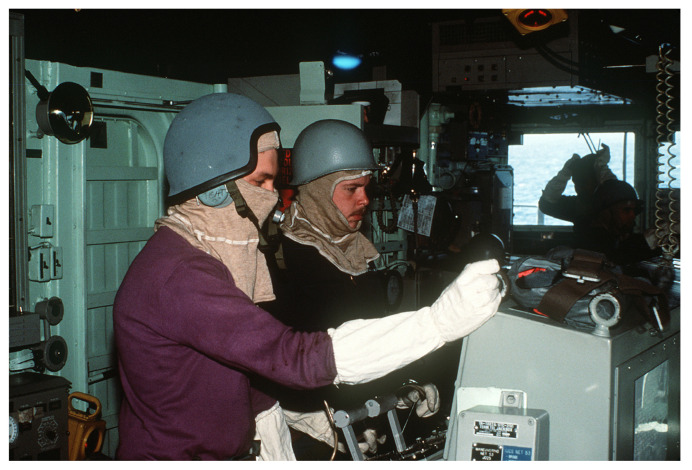
Sailors on the bridge of a U.S. Navy ship wear helmets and flash gear (flame resistant hood and gloves) as a general quarters drill is held during the multinational Rim of the Pacific exercise in 1990 (U.S. government photo, not in copyright).

Burn treatment advances developed during WWI included intravenous and subcutaneous fluid and electrolyte replacement, appropriate pain control during wound care, holistic critical care support, wound treatment based on burn depth, and advances in plastic surgery including the facial reconstruction of severe burns. In addition, based on injury pattern recognition, the Royal Navy introduced anti-flash clothing or “flash gear” consisting of flame-resistant hoods and gloves to be worn during high-risk naval operations (Figure 1). By World War II (WWII), the use of clothing to protect from thermal injury was standard practice [5,6,15,16,17].

### 2.2. World War II

During WWII, the lethality of naval warfare rapidly evolved. Instead of wooden sailing ships or armored battleships firing broadsides against enemy combatants, naval engagements were increasingly characterized by attacks from above and below the sea. At the beginning of the war, standard naval tactics focused on decisive battleship engagements, with carrier air power generally thought to have a supportive role. Following the decisive U.S. naval victories against Japan in 1942 at both Coral Sea and Midway, naval tactics rapidly evolved to center around the carrier strike group and the expanded use of naval airpower. The Battles of Coral Sea and Midway were the first naval battles where surface ships on either side did not sight each other during the engagement. These battles were won through the projection of naval airpower and the delivery of weapons of increasing kinetic energy and lethality such as bombs and torpedoes [8,18].

Comprehensive analyses of American shipboard casualties and injuries during WWII found that penetrating wounds (39%) were the most common injuries in survivors, followed by burns in 22–26% [19,20]. Air attacks accounted for 82% of shipboard burn injuries during naval action, with kamikaze attacks causing 71% and bombings 11%, with mean Killed in Action (KIA) rates of 37.4% and 52.4%, respectively. Burn injury rates in kamikaze attacks were 30% in survivors, 17% after bombing, 16.7% after torpedo attack, 15% from combined mechanisms, and 6.3% after a mine strike. Torpedo attacks were the most lethal, presumably due to the sinking of the attacked ship, resulting in a KIA rate of 66% [20].

By the end of WWII, American naval medical teams had become well-versed in the optimal management of severely burned patients, both immediately after injury and during periods of “days or weeks” before casualties could be transferred to a hospital ship or a well-resourced shore-based military treatment facility. Principles of naval burn care emphasized during WWII included controlling pain, burn shock resuscitation, limiting burn wound contamination, prevention of infection, and maintaining nutrition. For the U.S. Navy, the primary recommended resuscitation fluid in major burn injury became plasma. Freeze-dried plasma was “aboard all naval vessels… carried in large quantities”, and “ready for administration in five minutes time”. Nutritional support, prior to a patient’s ability to take in solid food, was provided via concentrated liquid feeding comprised of cocoa, powdered milk, eggs, simple syrup, and cod liver oils [21]. 

Rotary wing casualty evacuation did not become routine until the Korean War. During amphibious assaults and naval combat, casualties were moved from shore to ship, ship to ship, and ship to shore without helicopters. Patient movement from a shore with crashing waves to a ship or from ship to ship in rough seas is a dangerous activity. During the 1945 amphibious assault and Battle of Iwo Jima, approximately 21,000 casualties were evacuated off the island and treated on medically augmented troop transports and hospital ships and subsequently moved to higher echelons of care [22]. The U.S. and its allies have not managed a casualty burden of this magnitude, burn or otherwise, since WWII and needs to re-learn how to evacuate patients and re-supply medical supplies far forward on scale not seen since WWII without air superiority, including shore to ship, ship to ship, and ship to shore.

### 2.3. Current Shipboard Medical Tactics

**Figure 2:**
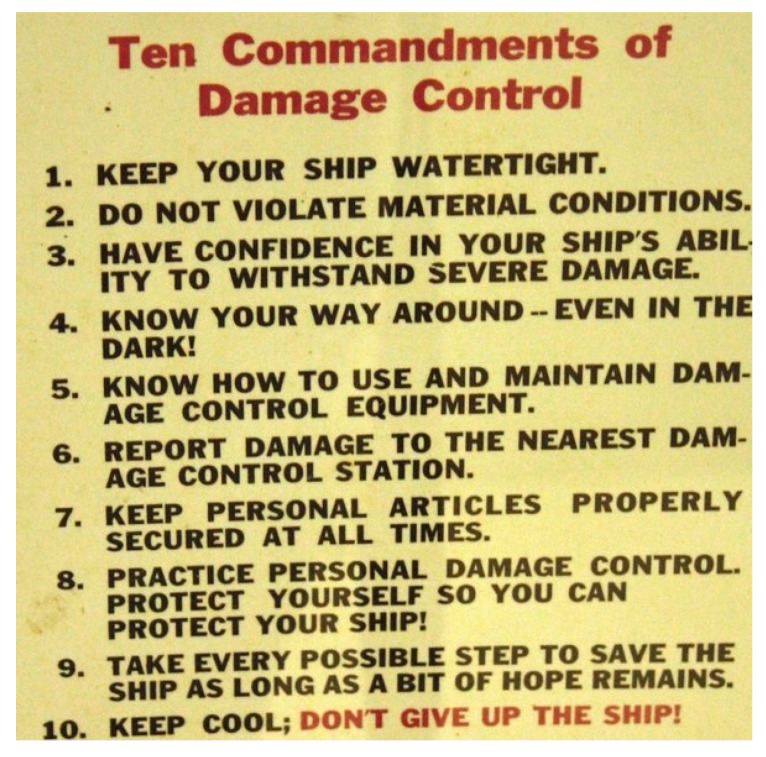
Ten commandments of damage control, U.S. Navy (photo not in copyright).

Current shipboard medical tactics have not significantly changed since WWII as the priorities at sea remain Ship, Shipmate, Self, in that order. The term ‘damage control’ was originated by the U.S. Navy to focus on saving the ship when threatened and formally became a rate during WWII in 1948. Damage control experts are on every warship to prevent accidents and are specifically trained in firefighting, ship stability, and chemical, radiological, and biological warfare defense [23]. Figure 2 displays the “Ten Commandments” of damage control, and the term has subsequently been indoctrinated into civilian and military medical lexicons. 

When a ship is threatened, focusing on damage control efforts to save the ship becomes the first priority; however, providing life-saving care to one’s shipmates so they can assist in these efforts will ultimately save the most lives and potentially enable the ship to maintain its fighting capability. Effective damage control is an all-hands priority and includes the correct use of equipment and various techniques to prevent or minimize damage to the ship from fire, explosion, battle, grounding, and loss of hull integrity from collision or other causes. Teams called “repair parties” are trained to perform specific tasks depending on the type of ship. Box 1 lists Damage Control Responsibilities common to all U.S. naval vessels. During naval action or when the ship is threatened, “General Quarters” is called to initiate the highest state of readiness on a ship. All hands are involved and all “repair parties” man their specific areas. Designated hatches and scuttles are closed to provide the greatest degree of subdivision and watertight integrity to maximize ship survivability. Crew movement about the ship is standardized, on the starboard (right) side of the ship the crew can only move forward in passageways and up ladders; on the port (left) side, movement through passageways is aft (towards the back of the ship) and down ladders. During General Quarters, all hands wear flash gear, button up uniforms, and tuck pants into their socks such that no skin is exposed [23].

Box 1 Universal Damage Control Team Responsibilities *. (* Adapted from Chapter 12, Damage Control in Navy Basic Military Requirements [23])

- Make repairs to electrical and communication circuits and restore power throughout the ship.
- Give first aid (e.g., TCCC) and transport injured personnel to battle dressing stations without seriously reducing the party’s damage control capabilities.
- Detect, identify, and measure radiation dose and dose rate intensities.
- Decontaminate the affected areas of nuclear, biological, and chemical attacks.
- Identify, control, and extinguish all types of fires.
- Control and remove flooding water (using various pumps). Hull integrity is typically controlled with various types of shoring materials which can include mattresses, pil-lows, canvas materials, hydraulic jacks, wooden wedges, beams, plugs, and blocks.
- Evaluate and correctly report the extent of damage in the repair parties’ area of re-sponsibility.
- Make emergency repairs to various piping systems.
- Be familiar with all damage control fittings in the assigned area, such as watertight doors, hatches, scuttles, ventilation systems, and various valves.
- Control and clean up hazardous material spills.

Tactical Combat Casualty Care (TCCC) principles (Table 1) should be followed during naval engagements and damage control efforts to save the ship. Therefore, any Sailor renders initial care at the point of injury [24,25,26]. Once the ship is no longer under threat and a safe treatment area can be established, standard TCCC algorithms can be enacted. This phase of care may or may not have a medical provider available and may last several hours. Timing and route of movement of medical providers to the patient and/or evacuation of the patient to medical spaces are dictated by the team within damage control central (DCC)—the central nervous system for all damage control efforts to save the ship. DCC receives input from the repair parties located throughout the ship and dictates the appropriate course of action based on the damage sustained. When the ship is mortally wounded, saving the ship is the priority over casualty care. DCC identifies safe routes through the ship and dictates *when* casualties can be moved [23]. Once patient movement is authorized by DCC, carrying litter-bound patients through narrow hatches or scuttles and up or down steep ladder wells can be a challenging and time-consuming evolution. Further triage, re-evaluation, resuscitation, treatment, and procedures can then occur at the main medical spaces or BDS. However, during a major mass-casualty event involving flame, smoke, and toxic fumes, designated medical spaces may be rendered unusable or the number of injured may mandate an alternate location. 

Previously, sailors were trained in basic first aid management of common injury patterns seen during WWII, including smoke inhalation, burns, electrical shock, jaw fractures, sucking chest wounds, abdominal evisceration, extremity fractures, and amputation [2]. It was not until 2021 that the U.S. Navy began integrating standardized TCCC training fleetwide, a process that is ongoing [24,25]. Table 1 lists recommended TCCC and burn interventions based on responder level [27]. While TCCC guidelines recommend Hextend as an alternative burn resuscitation fluid, it is currently not routinely available on most ships; lactated Ringer’s (preferred) and normal saline are.

## 3. Modern Shipboard Burn Injury during Routine Naval Operations

### 3.1. Major Burn Injury

Since WWII, burn mechanisms of injury continue to be common during routine naval operations. According to one analysis during the Cold War, between 1945 and 1988, there were 1276 known accidents involving 1533 ships in the major navies of the world, resulting in over 2800 deaths; 65% of these were in U.S. or Soviet Union personnel. U.S. Navy vessels were involved in 62.6% of accidents; 31.3% of all events involved ship collisions, and 33.9% involved some type of thermal injury mechanism including fire (18.3%), explosion (7.8%), ordinance mishap (3.7%), and ships propulsion casualties (4%), e.g., engines, boilers, or nuclear reactors [7]. Shipboard collisions can also result in thermal injury as evidenced when the larger aircraft carrier USS John F. Kennedy (CVN 67) collided with cruiser USS Belknap (CG 26) in 1975 off the coast of Sicily, causing fires and explosions on the smaller ship. Seven sailors were killed, including six on the *Belknap* and one on the *Kennedy* [7]. The 1989 collision of the USS Kinkaid (DD 965) with a civilian vessel off the strait of Malacca caused a fire in the starboard torpedo magazine. One sailor was killed due to blunt head trauma; injuries among survivors included inhalation of seawater or fuel (3), extremity fuel burns (2), fuel eye burns (1), open ankle fracture (1), and various contusions, sprains, and lacerations (9) [2].

During peacetime, a warship is most at risk to fire while in port during scheduled maintenance periods. Between 2008 and 2020, there were fifteen major fires on U.S. Navy vessels during maintenance periods, resulting in the loss of two vessels [28]. This was seen in the July 2020 loss of the USS Bonhomme Richard (LHD 6). One of the U.S. Navy’s eight most combat-capable amphibious assault ships (troop carrying warships designed to support ground operations during amphibious assaults with various aircraft and amphibious landing craft embarked), both in power projection and casualty receiving, the *Bonhomme Richard* was home ported in San Diego during a prolonged maintenance period when she caught fire after an explosion occurred in a lower vehicle storage compartment [18]. According to news reports and personal communication with the regional burn center at the time of the incident, 40 sailors and 23 civilians were injured fighting the fire, of which 21 were hospitalized, mostly overnight, with reported injuries of smoke inhalation and heat exhaustion. All patients were transported to mostly local civilian hospitals via civilian ambulances [29,30].

Though major fires during routine deployments are rare, they do occur. A comprehensive 50-year analysis of Naval Safety Command mishap data of all commissioned and active in-service U.S. subsurface and surface vessels (1970–2020) found that modern mishaps resulting in casualties, fatalities, and injuries decreased over the last 50 years. Of the 3127 total casualties identified, 35% involved some type of thermal energy, and 8.8% involved collisions causing blunt force and burn injuries. Table 2 demonstrates causes of burn injury and associated mortality from all U.S. Navy vessels (1970–2020), including 103 different fire, burn, or smoke inhalation events causing 923 casualties with a 13% mortality. Explosions and electrocution were the deadliest, with associated mortalities of 60.4% and 90.6%, respectively [4].

Approximately 8% of the U.S. Navy’s current surface and subsurface fleet strength provide a Role 2 (forward resuscitative surgical care) capability; the remaining 91.7% have Role 1 (non-surgical) medical capabilities. In 90% of Role 1 capable ships, the medical department is led by a non-physician Independent Duty Corpsman. Overall chemical, fire, and burn-related injuries occurring on these Role 1 capable ships were associated with a higher overall mortality compared to Role 2 vessels (19% vs. 8.3%, *p* < 0.05) [4]. While it is difficult to infer the cause, the clinical experience of Role 1 providers, limited supplies, reliance on crystalloid-based resuscitation, severity of thermal injury, and time/distance to a higher level of care could all be factors in this differential. No matter the cause, these findings warrant implementing improvements in burn injury care on smaller ships and increasing preventative measures. 

### 3.2. Minor Burn Injury at Sea

The true incidence of minor thermal injury in the deployed maritime environment is unknown, especially those burns primarily managed on deployment. The Benham et al. study reviewing 50 years of injury focused on reported mishaps that resulted in injury causing some type of disability, property damage, or loss of life; data on most minor injuries was not available [4]. A recent comprehensive analysis of burn injuries in deployed military service members from 2001–2018 did not specifically include events that occurred at sea [31]. However, one study of 196 U.S. Navy submarine patrols from 1997–1998 identified 915 medical events or injuries; of these, 5.6% were burns, and 3.2% were electrical injuries. The vast majority of burns (94%) occurred in food preparation areas, engine rooms, trash disposal units, and other mechanical areas. Electrical injuries occurred in engine rooms and other mechanical areas (65%) and food preparation areas (21%) [32]. In the author’s experience, these types of injuries typically occur in the hands or extremities. Those with minor burns to the hands and extremities can completely recover or develop a wide range of complications including minor functional impairment, complete functional limb loss, and amputation [31,33].

## 4. Modern Naval Warfare and Burn Injury at Sea

### 4.1. USS Stark and USS Cole Attacks

Between 1970 and 2020, there were six known attacks on U.S. naval vessels (Table 3). The 1987 Exocet missile attack on the USS Stark (FFG-31) and the 2000 watercraft-borne improvised explosive device (WBIED) attack on the USS Cole (DDG-667) resulted in significant damage to each ship, causing an overall mortality rate of 20%. Of those injured (112), mortality was 48%, demonstrating the lethality of these events in that one out of two servicemembers injured died of their wounds [2,3,34]. Of the survivors, 15.5% suffered burn injury and 10.3% asphyxia or inhalation injury. Other injuries included penetrating (6.9%) and soft tissue injuries (41.4%), fractures (24.1%), and traumatic brain injury (17.2%) [2].

Several characteristics of these two attacks are worth noting. Neither ship continued to be engaged in active combat after the initial strike and uncompromised air superiority allowed for rapid evacuation of casualties. The severely injured were evacuated off the *Stark* within 2–3 h via helicopter, and additional intravenous fluids and trauma supplies were needed and flown from nearby Bahrain. Smoke rendered the frigate’s medical department unusable, and the hangar was utilized as an alternate casualty triage site. Communications were immediately disrupted, requiring verbal communication to coordinate casualty movement throughout the ship. Among the survivors, specific burn injuries included two casualties with severe second- and third-degree burns; three sailors suffered flash burns. An unknown number of the ship’s firefighters suffered fatigue, heat stress, and smoke inhalation, rendering them ineffective. Personnel from two nearby U.S. ships were required to assist with damage control efforts; all fires were reported out 2 days after the attack [2,34]. 

The USS Cole WBIED attack was reminiscent of the fireboat attacks during the Age of Sail, except those driving this modern “fireboat” were on a suicide mission with no intention of jumping ship. While evaluating the potential threat of additional attack, the ship’s crew were able to simultaneously focus on damage control efforts to save the ship and conduct triage and casualty treatment. Of the thirty-seven injured survivors, four had first- and second-degree burns to the face or extremity, and three suffered toxic gas inhalation; two patients had pulmonary injuries, including blast lung and pulmonary contusions. Most of the injured (33) were off the ship within 90 min. The ship’s flight deck was utilized for casualty receiving and triage. While there was no walking blood bank capability on the ship, after a review of the ship’s medical records for blood type, several of the USS Cole’s sailors donated blood at local hospitals where their shipmates were being cared for [2,35,36,37].

### 4.2. Mine Warfare and the Power of Injury Prevention

Table 3 lists three different mine strikes events injuring less that 1% of the combined crews without any deaths. While most injuries were relatively minor, mine warfare can significantly damage and incapacitate a warship. Since WWII, 19 U.S. warships have been sunk or severely damaged, and naval mines accounted for 79%, more than missiles, bombs, and guns combined [38]. For the USS Samuel B. Roberts, personal protective equipment and the Commanding Officer’s (CO) actions prevented more severe injuries and death. While 5% of the crew were injured (Table 3), moments before the mine strike, crewmembers realized they were in a mine field, and the CO ordered “General Quarters”, requiring the crew to don flash gear and helmets (Figure 1). As a precaution, he also ordered the crew onto the upper decks as the ship slowly reversed out of the mine field. Unfortunately, a mine strike occurred, causing the ship to completely lose power; it was thrown up into the air, only to land in the water engulfed in a 100-foot fireball. The ship’s CO had to initially stop fighting the fire and turn the focus of the entire crew on damage control efforts to secure the hull breach and prevent the ship from sinking. Ultimately, four hours after the mine strike, the hole was plugged and the fire put out [39,40].

Of the 10 officially reported injuries, the CO suffered an ankle fracture and 7 sailors suffered burns; one was noted to be “severe”, about the face, neck, arms, and upper body. Unofficial reports suggest that an additional 57 crew members received minor injuries [3,4,40]. A review of the Naval Safety Command data by the authors (MDT) suggests that many more would have been severely injured had the crew not donned their flash gear and helmets and not been on the ship’s upper decks when the blast occurred, emphasizing the importance of injury prevention during naval warfare. 

### 4.3. The Falklands War

The most recent example of large-scale modern naval warfare during a peer or near-peer conflict was the 10-week-long Falklands War between Argentina and the United Kingdom (UK). The 1982 war over disputed South Atlantic territories is a relevant case study, albeit on a smaller scale, as naval medical providers prepare for a future “war at sea”. The Falklands War had a large UK maritime component involved in the denial of sea lanes and the conduct of amphibious assaults. Royal Navy warships were under constant threat of air attack by skilled Argentinian pilots; during the course of the war, 23 British ships were either lost or damaged. However, UK hospital ships were not under threat as Argentina did not target non-combatant vessels [41]. One analysis of ten warship attacks involving nine British and one Argentine warship during the conflict found that seven were either sunk or damaged beyond repair. The Argentine ship General Belgrano was sunk by a torpedo and eight of the British warships were attacked with a missile or bomb from the air, with one struck by a land-based missile [2,41]. In this cohort, 23.3% of the combined crews were injured or killed, with an overall mortality rate of 63.2% in those injured, with most dying instantaneously due to explosions, burns, or smoke inhalation and asphyxiation. The air bombing of the troop transport Galahad injured or killed 64% of the crew, with an overall mortality of 22.1%. Of surviving casualties, 49% suffered burn injuries to the face and hands; of these, 46 were evacuated nearly 8000 miles to the UK for further treatment [42,43]. Most of those injured during the war were eventually cared for on the hospital ship SS Uganda, including 666 “battle-related conditions” with smoke inhalation (12%) and hypothermia (10.3%) as common diagnoses. Of 516 actual “battle casualties”, 52.3% suffered penetrating wounds and 21% burns [43]. Most injured (and uninjured) survivors were off damaged ships, en route to further care, within minutes to 1 h, with 4 h being the longest time to evacuation [2,43,44,45].

## 5. Distributed Maritime Operations and the Future Fight

### 5.1. Distributed Maritime Operations

At the time of this writing, the risk of future Large Scale Combat Operations (LSCO) with peer or near-peer competitors on land and sea is ever present around the world. The Ukraine–Russia and Israel–Hamas wars are ongoing, and the Chinese military is a significant pacing threat to the U.S. and its allies. The People’s Liberation Army Navy continues to grow and has an estimated current fleet strength of 370 ships and submarines, including three aircraft carriers [46], whereas the U.S. Navy currently has an active fleet of 238 surface and subsurface vessels [47]. To prepare for future LSCO, U.S. and North Atlantic Treaty Organization (NATO) military strategy is coalescing around the concept of Multi-Domain Operations, where military operations will be coordinated and orchestrated across vast distances in the sea, land, air, space, and cyberspace domains. Previously, operations in these domains either did not exist (e.g., space and cyberspace) or occurred largely independently. Included within this concept are Distributed Maritime Operations (DMO) for the Navy and Expeditionary Advanced Base Operations for the U.S. Marine Corps. During DMO, naval forces will be geographically distributed but integrated through an architecture of new and developing technologies to synchronize operations across all domains [48]. The reason for DMO is partly based on a defensive posture intended to counteract the significant missile firepower of peer competitors, complicating targeting and enhancing survivability by dispersing the force over large geographic areas. Simultaneously, during DMO, naval assets will launch “massed volleys of networked weapons to overwhelm adversary defenses” [49]. DMO will allow naval warfare to be executed at the fleet level instead of by singular carrier strike groups or amphibious ready groups. Expeditionary Advanced Base Operations is a warfighting concept developed by the U.S. Marine Corps to support and integrate with DMO by deploying small-footprint expeditionary forces from the sea to austere inshore and ashore contested or potentially contested locations. 

Practically speaking, these new warfighting doctrines supporting LSCO will change the logistics of and how combat casualty care is performed. Forward deployed caregivers will have to manage patients for prolonged periods of time at each role of care. For example, the concept of Prolonged Casualty Care (PCC) and clinical practice guidelines have been developed to guide Medics and Corpsman managing patients with limited resources for prolonged periods of time beyond initial TCCC [50,51]. Role 2 caregivers providing Forward Resuscitative Surgical Care will likely have to perform more definitive surgical care beyond initial damage control surgery techniques to control hemorrhage, restore perfusion (e.g., temporary vascular shunts), control contamination, and achieve temporary abdominal closure. Instead of moving casualties rapidly to higher echelons of care, austere Role 2 critical care for prolonged time periods will also likely be required [52,53]. As will robust en route care (ERC) capabilities to move patients over significant distances within and between multiple domains. 

### 5.2. The “Carrier Killer” Anti-Ship Ballistic Missile

Recently, the People’s Republic of China’s modernization and expansion of its fleet, coupled with the construction of strategic defenses in the South China Sea, has garnered increased press attention. While it is unknown if China’s hypersonic anti-ship ballistic missile (ASBM), the so-called “carrier killer” weapons system with a range of 2500 miles, could successfully target an aircraft carrier, the over-the-horizon threat to U.S. capital ships is becoming increasingly real [54]. If carrier air power has truly been replaced by ASBMs, the distance to execute naval warfare in the air domain has significantly increased since WWII. With the increased lethality of these weapons systems comes the risk of a large number of casualties with blast and burn-related injuries not seen since WWII. Current medical and surgical capabilities after this type of attack will be woefully insufficient; therefore, investments should focus on both tactical injury prevention and force-enabling medical capabilities. 

While the last major attack against an aircraft carrier occurred during WWII, four aircraft carrier fires since then are worth reviewing because they either involved ordinance-related mishaps or massive explosions (Table 4). Five to eight percent of the crews were injured, with a mortality rate ranging from 8% to 51%. On the USS Bennington, 1 of 3 physicians and 1 of 22 corpsmen were killed in the blast [55]. The USS Forrestal fire was the first major fire to occur on a U.S. supercarrier and the forward hangar bay and mess decks were used for casualty triage. Additional medical supplies had to be flown in from nearby ships, and medical evacuations were delayed due to the damaged flight deck [54,56]. During the USS Enterprise fire, 18 explosions were reportedly heard and 15 aircraft were destroyed [57].

### 5.3. Clinical Vignette: Severe Maritime Burn Injury and the Tyranny of Distance

In July 2022, during the 26-nation Rim of the Pacific (RIMPAC) naval exercise, a boiler room fire occurred on a coalition nation corvette, severely injuring two sailors (86% and 70% total body surface area cutaneous burns, both with inhalation burns). Multiple assets, including the U.S. Coast Guard, the French Navy, and the U.S. Navy, coordinated to rescue and evacuate these patients. This included an ad hoc ERC team, including a Critical Care Nurse, Certified Nurse Anesthetist, and Search and Rescue Medical Technician, who were mobilized from a nearby U.S. aircraft carrier. One burn casualty required intubation during transport; the other was intubated shortly upon arrival to Tripler Army Medical Center in Hawaii. Both required ongoing resuscitation including vasopressor support during evacuation. 

Transport time from point of injury to the U.S. Army Institute of Surgical Research (USAISR) Burn Center took 11 days, with a total of seven handoffs. At the USAISR, the mean hospital length of stay (LOS) was 101 days, the intensive care unit LOS was 50 days, and the number of ventilator days was 10.5. They underwent an average of 14 operations focused on excision and grafting of burn wounds and the correction of eyesight-threatening scarring of the periocular structures. They were transfused an average of 78 units of blood products. As inpatients, they received an average of 4.4 h of direct rehabilitation per day, and as outpatients, they received an average of 63 rehabilitation treatment sessions. After 5.5 months, these sailors successfully returned to their home country. These data underscore the complex, resource-intensive nature of burn care from point of injury through definitive care.

The RIMPAC boiler room mishap, the Falklands War, and the *Cole* and *Stark* attacks demonstrate that shipboard medical caregivers have been accustomed to robust medical support through both rapid medical evacuation and rapid augmentation of additional supplies or providers [2]. During current and future contested DMO, rapid medical evacuation is unlikely to be available. Shipboard caregivers will be required to manage critically ill, burned, and injured patients for prolonged periods of time of likely days to weeks, a paradigm of combat casualty care not seen since WWII. Medical care will be a limiting factor in any future scenario, and therefore robust investments in prevention and countermeasures must be undertaken in addition to efforts focused on improving clinical care in these potential horrific wartime situations. 

## 6. Preparing for a Future War at Sea: The Good, the Bad, and the Ugly

Since the end of WWII, austere burn care at sea has been experiencing a significant “Walker Dip” [14]. To prepare for the potential of a large number of burned casualties during a future war at sea, Military Medicine has several resources (The Good), opportunities for improvement (The Bad), and urgent priorities (The Ugly) to consider. What follows is an assessment of each. 

### 6.1. The Good

#### 6.1.1. Clinical Practice Guidelines

Various resources are available to help prepare Role 1 and Role 2 maritime caregivers to manage critically ill patients with thermal injury [1,26,58,59,60]; the most important and readily available are The Joint Trauma System Clinical Practice Guidelines (CPG). Box 2 lists the CPGs that describe optimal burn injury management across all roles of care, including Role 1 prolonged casualty care.

Box 2 **List of CPGs describing optimal deployed burn injury management.**

- Tactical Combat Casualty Care (TCCC) Guidelines available at: https://books.allogy.com/web/tenant/8/books/b729b76a-1a34-4bf7-b76b-66bb2072b2a7/ (accessed on 31 July 2023).
- Burn Care—CPG ID: 12 available at: https://jts.health.mil/assets/docs/cpgs/Burn_Care_11_May_2016_ID12.pdf (accessed on 31 July 2023).
- Inhalation Injury and Toxic Industrial Chemical Exposure—CPG ID: 25 available at: https://jts.health.mil/assets/docs/cpgs/Inhalation_Injury_Toxic_and_Industrial_Chemical_Exposure_26_Jul_2016_ID25_updated.pdf (accessed on 31 July 2023).
- Burn Wound Care in Prolonged Field Care (PFC)—CPG ID 57 available at: https://jts.health.mil/assets/docs/cpgs/Burn_Management_PFC_13_Jan_2017_ID57.pdf (accessed on 31 July 2023).
- Austere Resuscitative Surgical Care (ARSC)—CPG ID: 76 available at: https://jts.health.mil/assets/docs/cpgs/Austere_Resuscitative_Surgical_Care_30_Oct_2019_ID76.pdf (accessed on 31 July 2023).
- Prolonged Casualty Care Guidelines (PCC)—CPG ID 91 available at: https://jts.health.mil/assets/docs/cpgs/Prolonged_Casualty_Care_Guidelines_21_Dec_2021_ID91.pdf (accessed on 31 July 2023).

#### 6.1.2. US Army Institute of Surgical Research Burn Center

The USAISR Burn Center, Fort Sam Houston, is an excellent resource for deployed military providers, providing both synchronous and asynchronous consultations for patients with minor burn injuries and to assist with resuscitation and management during prolonged holding. The USAISR Burn Center was also where the aforementioned sailors involved in the RIMPAC boiler room mishap ultimately received both in-patient and out-patient burn and rehabilitative care. Additionally, some surgeons and/or members of U.S. Navy Fleet Surgical Teams (typically deploy on amphibious warships) have had the opportunity for just-in-time clinical experiences at the USAISR Burn Center, but this practice is not routine across maritime surgical teams.

##### USAISR Burn Center contact information:

- DSN 312-429-2876 (429-BURN)
- Commercial (210) 916-2876 or (210) 222-2876
- Email to burntrauma.consult.army@health.mil

#### 6.1.3. Operational Virtual Health Consultation Resources

**Figure 3:**
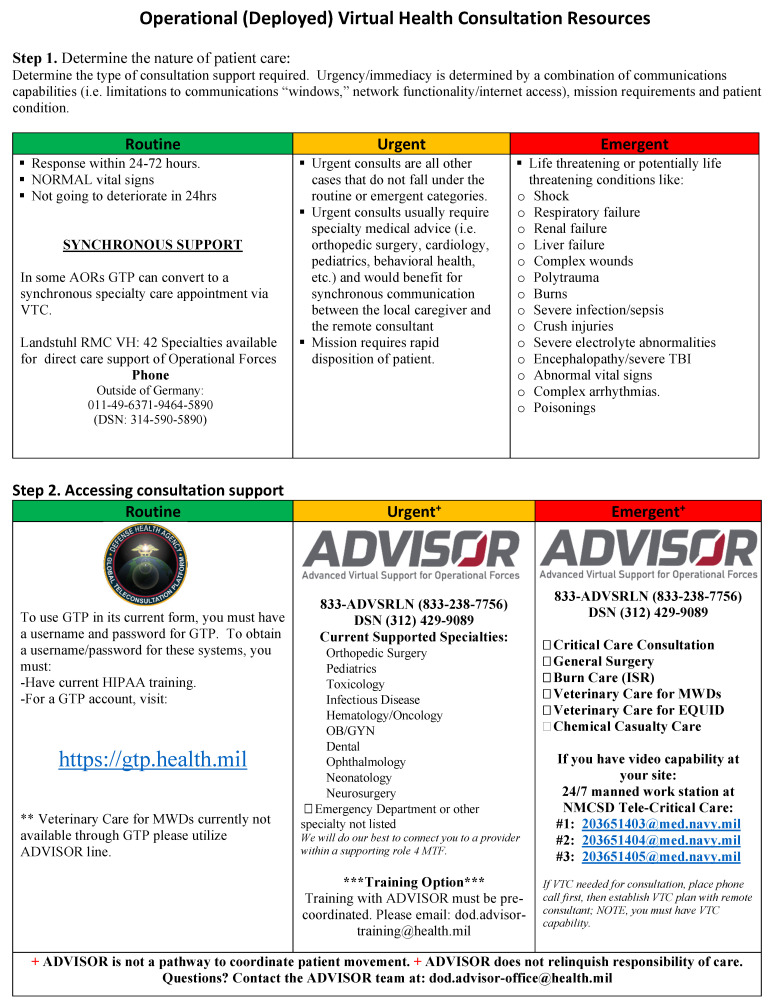
ADVISOR synchronous and asynchronous contact instructions. (Source: Military Health System (MHS) Virtual Medical Center, used with permission). ADVISOR—Advanced Virtual Support for Operational Forces, AOR -area of responsibility, GTP -Global Teleconsultation Portal, VTC -video teleconferencing, RMC -Regional Medical Center, VH -virtual health, TBI -traumatic brain injury, HIPAA -Health Insurance Portability And Accountability Act, MWD -military working dog, OB/GYN -obstetrics and gynecology, ER -emergency room, ISR -Institute of Surgical Research; NMCSD -Naval Medical Center San Diego.

The Advanced Virtual Support for Operational Forces (ADVISOR) is also an excellent resource for both synchronous and asynchronous sub-specialty consultation, including critical care and the USAISR Burn Center (Figure 3). However, during contested DMO or naval engagement in a future war, synchronous or asynchronous clinical support is not likely to be available when they are needed the most.

#### 6.1.4. Military Civilian Partnerships

Military Civilian Partnerships (MCP) also provide an opportunity for rotating and embedded providers to obtain clinical burn experience. Current Navy Medicine strategic partnerships with Stroger Hospital of Cook County in Chicago, Los Angeles County and University of Southern California in Los Angeles, and Penn Medicine in Philadelphia, all either have burn units or are developing affiliations with regional burn units providing embedded and rotating providers the potential for burn clinical experiences. United States Marine Corps Role 2 surgical team members (comprised of U.S. Navy caregivers) stationed in Southern California have the opportunity for burn care clinical rotations and training through regional MCP with both University of California Irvine and the University of California San Diego. 

### 6.2. The Bad

#### 6.2.1. Availability of Routine Pre-Deployment Burn Care Training and Clinical Experience

While the USAISR Burn Center and aforementioned MCPs provide the potential of clinical burn experiences for Role 2 providers supporting worldwide deployable Fleet and Marine Corps expeditionary and maritime platforms, these experiences are the exception, not the rule. Burn clinical care experiences are not part of routine pre-deployment training cycles across the naval force. Nor is Advanced Burn Life Support (ABLS) or an equivalent military-specific burn curriculum a pre-deployment requirement for Role 1 and 2 naval caregivers. Relevant clinical experiences in Burn, Trauma, and Critical Care and courses such as ABLS must be requirements to prepare Role 1 and 2 providers to manage critically ill, burned, and injured patients in austere maritime environments [2].

#### 6.2.2. Distribution and Re-Supply of Crystalloid and Dressing Supplies

As demonstrated by the USS Stark missile attack and the USS Forrestal fire, additional medical supplies, particularly crystalloid intravenous fluids, were needed very quickly and transported by air from nearby vessels. For larger warships, there may be more space for additional crystalloid fluids and dressing supplies, but smaller surface ships, and submarines in particular, have very limited space. Novel logistics solutions are needed, such as drone technologies providing re-supply of crystalloids and other supplies in the air and maritime domains (surface and subsurface). 

### 6.3. The Ugly

#### 6.3.1. Clinical Skills Sustainment Opportunities for Independent Duty Corpsmen 

Unique to naval service and crucial to deployed maritime casualty responses, Independent Duty Corpsman (IDC) are senior enlisted caregivers who provide protocol-based clinical care under distant physician supervision in austere environments on land and sea. During their initial IDC training, they receive basic burn care didactic instruction and non-standardized procedural cadaver-based instruction. However, after initial training, they receive no regular clinical skills sustainment experiences beyond primary care or training beyond regular TCCC didactics and skills to prepare them to manage burned, injured or critically ill patients for the days to weeks that will be required during a future war at sea. These versatile caregivers are at the tip of Navy Medicine’s spear, leading 90% of the Role 1-capable submarine and surface ship medical departments, highlighting their critical role in the maritime trauma system and the importance of implementing routine advanced procedural skills training and clinical skills sustainment relevant to Burn, Trauma, and Prolonged Casualty Care (e.g., critical care) [2,4]. IDCs will also need to be given the supplies (e.g., nasogastric tubes (NGT), electrolyte solutions) and appropriate training to perform coached oral or NGT enteral rehydration, given the limited ability to store crystalloids on smaller vessels.

#### 6.3.2. Acute Burn and Hemorrhagic Shock Resuscitation: Plasma and Whole Blood Availability

Thermal injuries during war on land or sea generally do not occur in isolation [31,59], and combined burn and traumatic injuries have a significantly higher mortality and a greater likelihood for associated inhalation injury [61,62]. Therefore, these patients require an organized initial physiologic-based assessment, treating the greatest threat to life first. This includes performing hemorrhage control, initiation of blood product resuscitation, and traumatic injury management before formally addressing the burn injury while being cognizant of the fluid resuscitation needs attributable to the burn [24,60]. As such, blood product availability is a major opportunity for improvement on American naval vessels. Only 17.4% of all active in-service USS ships and submarines have stored blood products or walking blood bank (WBB) capability; submarines have none [4]. However, during WWII, stored fresh whole blood (drawn from the ship’s crew prior to engagement using standardized protocols) and freeze-dried plasma were readily available to resuscitate injured and burned casualties during naval engagements. Those concerned with the feasibility of either whole blood or dried plasma during war at sea need only to look at the allied experience in WWII, where both were ubiquitous and readily available in the European and Pacific Theatres [2,21,63,64,65]. Over the last twenty years, Military Medicine re-learned how efficacious, life-saving, and feasible whole blood, either fresh from walking blood banks or stored low-titer type O whole blood (LTOWB), is during hemorrhagic shock resuscitation [66,67,68,69,70,71,72,73,74,75]. Civilian studies have also consistently demonstrated LTOWB to be associated with improved or non-inferior outcomes in both pre-hospital and hospital settings [76,77,78,79,80,81,82,83,84,85,86,87]. Thankfully, in the recent past, severe burn injuries have not accounted for a significant proportion of combat casualties [31]. The feasibility and efficacy of dried plasma resuscitating burned naval casualties during WWII may be analogous to re-learning the value of whole blood resuscitation. If so, this will hopefully be re-learned *before* Military Medicine must care for large numbers of burn casualties again. 

From a physiologic standpoint, colloids limit the “fluid creep” that can occur during burn resuscitation by limiting edema formation in non-burned tissues via re-establishing intra-vascular colloid oncotic pressure and decreasing fluid flux. This contributes to reduced resuscitation volumes in clinical studies and restores cardiac output faster in experimental models [88,89]. Last updated in 2016, the current Joint Trauma System Burn Clinical Practice Guideline recommends considering the use of 5% albumin or Fresh Frozen Plasma (FFP) in the first 48 h of acute burn resuscitation if the hourly IV crystalloid rate exceeds 1500 mL/h, or for persistent oliguria and/or hypotension [90].

The pendulum of data and opinions regarding the use of plasma and albumin for acute burn resuscitation has swung back and forth. There were previous concerns about a higher mortality associated with albumin, but this has not been seen in more contemporary studies; experts now recommend high-quality modern studies to definitively answer the question [91,92,93,94]. A 2009 survey found that nearly 1/3 of responding international burn centers utilized colloids, with nearly 50% starting in the first 24 h of resuscitation; 14% using FFP and 20.8% albumin [95]. Currently, albumin tends to be used in more severely injured burn patients. The 2023 prospective observational “ABRUPT” trial found that patients in North America resuscitated with albumin tended to be older with more severe burn injury and organ dysfunction at presentation, and were more likely to develop abdominal compartment syndrome, require extremity fasciotomies, have renal replacement therapy initiated, and require longer mechanical ventilation. Those resuscitated with plasma were excluded from the study [88]. In trauma patients without burns, albumin resuscitation has been associated with a significantly higher mortality in patients with severe traumatic brain injury [96,97]. Given this and the uncertainty of albumin in acute burn resuscitation, many military providers advocate for freeze-dried plasma use in deployed environments on land and sea [65,98].

One concern regarding FFP resuscitation in injured and critically ill patients is the complications associated with its use, particularly when not part of a balanced resuscitation strategy during massive transfusion for hemorrhagic shock resuscitation. These include increased infectious and inflammatory complications such as sepsis, pneumonia, venous thromboembolism, acute respiratory distress syndrome, and multi-organ dysfunction syndrome. Despite this, the use of plasma in trauma patients confers a mortality risk reduction for every unit transfused [99,100,101,102].

While promising, very few high-quality studies describing outcomes using FFP in acute burn resuscitation are available. Small clinical studies demonstrate that FFP-based resuscitation is associated with smaller resuscitation volumes and is less likely to put patients at risk for abdominal compartment syndrome or acute kidney injury requiring renal replacement therapy [103,104]. Improved mortality compared to crystalloid-only and albumin-based resuscitation strategies have also been demonstrated [104,105]. However, inflammatory complications have been observed. In a retrospective analysis of 18 patients (mean TBSA 55.1%) resuscitated with FFP, one without inhalation injury developed Transfusion-Related Acute Lung Injury within six-hours of completing FFP transfusion [106].

Only twenty-one U.S. Navy warships and its two hospital ships routinely carry FFP during deployments: nine large amphibious warships (2 LHA and 7 LHD) carry 50 units, twelve amphibious transport dock (LPD) ships carry 5 units, and the two hospital ships each carry approximately 110 units [107,108]. Logistically, it would be challenging to have ubiquitous FFP availability throughout the fleet. Given the relative uncertainty of albumin’s impact on mortality and complications in burn patients [92,105] and its association with high mortality in traumatic brain injury [96,97], many feel that dried plasma is safer than albumin and more feasible than FFP in austere operational environments for acute burn resuscitation [65,98]. Dried plasma has several advantages that make it ideal for the deployed maritime environment in both the treatment of burn and hemorrhagic shock:

- It can be stored at room temperature for up to two years and pre-positioned in ship Battle Dressing Stations [98].
- It can be rapidly reconstituted in sterile water for administration [98].
- It stabilizes the endothelium, treating the endotheliopathy of burn shock [65,89,109].
- It minimizes over-resuscitation with crystalloids [65,104].
- It decreases the risk of complications associated with “fluid creep”, including abdominal and extremity compartment syndromes [65,103].
- As many burn patients in the military setting have concomitant traumatic injuries, it also provides immediate resuscitation for hemorrhagic shock until other blood products are available [98].

The U.S. stopped producing pooled dried plasma in the 1950s because of the viral hepatitis transmission risk. Now, it is routinely used safely in several countries by both civilian and military emergency medical systems, including in Canada, the Czech Republic, France, Germany, Israel, Norway, South Africa, and the UK. Dried plasma products are currently manufactured with regulatory approval by the French Military Medical Service, the German Red Cross, and the National Bioproducts Institute of South Africa. While French freeze-dried plasma has been available to U.S. Special Operations Command since 2011 (through an emergency use authorization), it is not pre-positioned or available for resuscitation in the amounts that will be needed during LSCO [98].

In summary, high-quality data is needed to definitively answer the question regarding which colloid (plasma or albumin) should be utilized in acute burn resuscitation. Given that burn injuries generally do not occur in isolation during naval combat, and the uncertainty of albumin resuscitation in traumatic brain injury and acute burn resuscitation, the logistical advantages of dried plasma coupled with its versatility in both hemorrhagic shock and acute burn resuscitation make it an ideal resuscitation colloid in the deployed maritime environment.

#### 6.3.3. Austere Role 2 and En Route Critical Care Capability

Role 2 critical care capability for LSCO is another major gap. Resuscitating and treating burn casualties is time-, labor-, and supply-intensive, requiring proficiency in core critical care skills (Box 3) [2,110]. Currently, most maritime surgical team providers do not receive experience managing burn or critical care patients beyond their initial training and certification. This is further complicated by the fact that the American Board of Surgery no longer requires a burn rotation during surgical training. Often, the military caregiver with the most regular critical care experience is the CCRN, although their exposure to burn care is highly variable and often non-existent. The average American general surgery resident receives variable exposure to surgical critical care and burns: 3 and 0.8 months, respectively [111]. Given the well-documented decreasing surgical workload at all U.S. military hospitals [112], it is highly unlikely that any Navy caregiver is maintaining robust burn and critical care proficiency if they are only sustaining clinical skills at their assigned military hospital.

Box 3 **Key Role 2 critical care and burn competencies [90,110].**

Respiratory Support

- Non-invasive and invasive airway management (e.g., intubation and cricothy-roidotomy).
- Non-invasive and ventilator management, airway maintenance, oxygen delivery, and monitoring. Consider and manage inhalation injury.

Resuscitation and Hemodynamics

- Intravenous and central venous access.
- Shock management, vasopressors, monitoring, and oxygen delivery.

Burn Resuscitation

- Rule of Tens: 10 mL/h × % TBSA. Increase fluid rate by approximately 20–25%/h to maintain a urine output (UOP) of 30–50 mL/h. If UOP >50 mL/h, decrease fluid rate by 20% for two consecutive hours.

Burn Wound Management

- Calculate burn size using Lund and Browder chart. Clean and debride wounds if possible. Wrap burns (scalp, trunk, neck, extremities) in sterile gauze soaked with a 5% solution of Sulfamylon. Alternatively, burns may be dressed with sil-ver-impregnated nylon, covered with sterile gauze, and moistened with sterile water (this can be left on for as long as 7 days).
- In patients who cannot be evacuated for burn excision, as a bridge to surgical care, consider using silver sulfadiazine cream alternated twice daily with mafenide acetate (Sulfamylon) cream to provide antimicrobial penetration of thick burn eschar.

Nutritional Support

- Early and continuous nutrition is vital to wound healing. Patients who are able to eat may need supplementation to meet calorie goals. Provide approximately 35 kcal/kg/day to burned adults. Nasoenteric feeding should be high protein, low fat.

Complication Prevention and Management

- Venous thromboembolism, infection, pressure ulcers, lines/tubes.

Transportation

- En route care preparation.

As the 2022 RIMPAC boiler mishap demonstrated, a U.S. Navy ad hoc ERC with the appropriate airway, respiratory, and shock management skills including intubation capability had to be mobilized to provide immediate airway management and resuscitation during evacuation. The U.S. Navy has begun implementing an organic two-person ERC capability designed specifically to support DMO, comprised of an emergency medicine nurse or critical care nurse and a search and rescue medical technician. However, had one of these teams been available to transport the burned sailors from the RIMPAC mishap, they likely would not have had the appropriate training and skills to perform emergency intubation. For this reason, some have advocated for a more capable team analogous to the UK’s medical emergency response team or the U.S. Air Force Critical Care Air Transport Team (CCATT) that are physician-led and can provide key critical care competencies on rotary wing or other casualty evacuation platforms [2,113,114,115]. Beyond just providing robust critical care during ERC, the ability to augment maritime Role 2 surgical teams when and where they are needed may prove more beneficial. Others have advocated for a U.S. Naval (Navy and/or Marine Corps) modular maritime rotary wing critical care transport capability both for ERC and for rapid augmentation of shipboard Role 1 and Role 2 teams during DMO [2]. This scalable, modular capability should include the ability to provide physician-led key critical care competencies and capabilities (Box 3). Training, equipment, and supplies to provide continuous renal replacement therapy (or peritoneal dialysis) augmentation or during ERC via rotary wing may also be required for the most critically ill burn and combat casualties, as was routinely performed during CCATT aeromedical evacuation during the last twenty years of war [116,117]. While routine evacuation by air will likely not be routinely possible during LSCO, there will be windows of opportunity. The ability to rapidly provide critical care capability where and when it is needed could save many lives.

#### 6.3.4. Burn Injury Prevention at Sea

The vulnerabilities of sailors on a ship engaged in combat are innumerous, and the maritime environment provides a potential scenario for multiple/mass casualties with limited materiel and personnel resources. While clinical training, experience, and burn education must be improved, concurrent investments in prevention and countermeasures to decrease the likelihood and damage of these attacks must be continuous as well. As the mine strike on the USS Samuel B. Roberts demonstrated, preventative measures save lives. Throughout the 20th century, advances in personal protective equipment including flash gear, fire retardant shipboard uniforms, and helmets (Figure 1) have prevented injury and death from thermal, blast, and blunt mechanisms of injury during naval warfare. However, given the high-energy threat of “carrier killer“ anti-ship ballistic missiles combined with the metallurgy of modern ships, new 21st century advances are needed in developing personal protective equipment, perhaps even maritime body armor designed to prevent thermal and blast injury during a future war at sea.

#### 6.3.5. Burn Mass Casualty, Triage and the Prevention of Moral Injury

Finally, those responsible for patient triage and resource management may be confronted with difficult decisions related to limitations of care. These decisions must be based upon available reliable information, including number of injured, access to treatment areas, ongoing threat assessments, and evacuation details. If the U.S were to aid in the defense of Taiwan against a Chinese invasion, a recent unclassified wargame estimates there will be between 6960 and 10,000 U.S. casualties in the first three–four weeks alone, with at least 3200 killed in action; this includes two aircraft carriers (CVN) and 7–20 surface ships, including Cruisers (CG) and Destroyers (DDG), lost [118]. Table 5 demonstrates the estimated casualties if each ship in two Carrier Strike Groups (1 CVN, 2 CG, 2 DDG each) and one Surface Action Group (1CG, 2 DDG) were struck by at least one anti-ship ballistic missile. Casualty estimates are based on the USS Stark missile strike and the attack on the USS Franklin, the most devastating aircraft carrier attack of WWII [34,119]. To estimate the percentage of burn casualties in survivors, 30% was used based on historical WWII surface ship kamikaze attacks [20]; of the 3021 surviving casualties, 908 are predicted to have some type of burn injury. Juxtaposed with each ship’s medical capabilities (Table 5), it is easy to see how quickly caregivers will be overwhelmed; even more so if medical spaces are damaged in the attack. It should be noted that all ward beds on warships are stacked, therefore, 50% are minimal access; patients on the top bunk must be able to climb up. In this hypothetical scenario, challenging decisions will be required, and they must be based on available resources and the foundations of medical ethics. In order to save those who can be saved with the available resources, patients who would normally survive their burns or injuries may not. Leadership must address the potential negative impact on the entire care team that can result from such decisions before deployments and real time during deployment. Pre-deployment ethics training [120,121] and mass-casualty exercises incorporating scenarios with expectant injury patterns may blunt provider moral injury during real maritime mass-casualty events. Daily team debriefings, monitoring for signs of stress, and providing rest time may also help mitigate some of these effects.

## 7. Back to the Future: Conclusions and Recommendations

With the over-the-horizon specter of a future multi-domain war with near-peer or peer competitors involving DMO, it is time to re-learn the clinical and logistical lessons earned in blood and treasure during WWII before it is too late. To prepare for burn injury, mass-casualty incidents, and associated concomitant injuries, the following actions are recommended (Box 4).

Box 4 **Recommendations to prepare for burn care during a future war at sea.**

- Implement regular Advanced Burn Life Support training or equivalent to all Navy Medicine providers, including Independent Duty Corpsmen [2].
- Role 1 and Role 2 providers must be provided routine clinical skills sustainment experience relevant to Burn, Trauma, and Critical Care, including a focus on spe-cific skill sets such as airway and ventilator management, resuscitation, sedation, and critical care procedural skills [2].
- Explore the feasibility of a rotary-based, physician-led critical care transport and augmentation capability [2,113,114,115].
- Develop and implement a comprehensive whole blood capability across all Role 1 capable surface and subsurface vessels [2].
- Re-develop and implement a dried plasma capacity and capability throughout Navy and Military Medicine [65,98].
- Improve clinical documentation for patient care as well as submitting the clinical data from shipboard injuries to the Department of Defense Trauma Registry [4].
- Research and development efforts should focus on clothing, helmets, and perhaps “maritime body armor” to prevent thermal and blast injury from high-energy weapons systems.

First, forward-deployed maritime Role 1 and 2 caregivers must receive formal training analogous to ABLS at *regular intervals* similar to TCCC and other trauma pre-deployment training. However, ABLS does not cover care past the first several hours post-burn, assumes that transfer to a U.S. burn center will be rapid, and that the ability to communicate between initial medical personnel at the point of injury and burn specialists at a receiving burn center is readily available; thus, training in burn injury prolonged casualty care past the first 24 h is needed for selected personnel. It is important to recognize that there is currently a gap between the number of available ABLS courses in the U.S. and the number of military personnel who require ABLS training. Forward deployed maritime caregivers must also be provided regular clinical experiences relevant to Burn, Trauma, and Critical Care including training in key procedural skills (Box 3). In addition to the current Navy ERC teams being implemented, the authors recommend exploring the feasibility of a scalable rotary wing physician-led critical care augmentation capability to provide organ support and resuscitation for critically ill burn and trauma patients.

Whole blood and dried plasma must be as readily available to modern Role 1 and 2 shipboard providers as it was during WWII [2,21,63,65,98]. Plasma, particularly freeze-dried plasma, potentially represents the most efficacious burn resuscitation fluid in an austere environment characterized by prolonged patient evacuation and delayed resupply. Given the concomitant penetrating and blunt injuries associated with wartime burns, a comprehensive whole blood capability should be implemented across all Role 1 capable surface and subsurface vessels. In combat casualties, rapid access to whole blood saves lives [69,122,123] and will allow some injured sailors to return to the fight or damage control efforts to save their ship. Military Medicine must go back to the future; redeveloping whole blood and dried plasma capabilities and capacity throughout the naval force must be an urgent priority.

While the Navy Safety Command tracks mishaps at sea and their databases contain rudimentary injury information, there is no current mechanism for clinical data from shipboard injuries to be submitted to the Department of Defense Trauma Registry [4]. Navy Medicine, the Naval Safety Command, and the Joint Trauma System must partner to routinely track and analyze clinical shipboard injury data. With clinical data and rapid performance improvement, gains in care as well as education and training will be made in a rapid, data-driven fashion. Finally, given the raw destructive power of modern high-energy weapons, research and development efforts should focus on thermal and blast injury prevention and mitigation of effects due to these weapons systems. The combination of robust clinical data collection, rapid process improvement, and thermal injury prevention will improve survivability and enhance the lethality of the Naval force during routine operations and a potential future war at sea.

## Figures and Tables

**Table 1:** Tactical Combat Casualty Care (TCCC) interventions by responder level *.

Responder Level	Provider Type	TCCC Skill Examples	TCCC Burn Injury Intervention
Tier 1	All service members	Basic life-threatening assessment to include hemorrhage, airway, and respirations. Basic hemorrhage control (e.g., direct pressure, packing, extremity tourniquet) Basic airway maneuvers (e.g., sit up/lean forward, jaw thrust) Care under fire/threat	Stop the burning process. Basic burn assessment: assess and treat as a trauma casualty with burns and not burn casualty with injuries. Facial burns, especially those that occur in closed spaces, may be associated with inhalation injury. Aggressively monitor airway status and oxygen saturation in such patients. Estimate total body surface area (TBSA) burned to the nearest 10% using the Rule of Nines. Apply dressing to burns: cover the burn area with dry, sterile dressings. All TCCC interventions can be performed on or through burned skin in a burn casualty. Burn patients are particularly susceptible to hypothermia. Extra emphasis should be placed on barrier heat loss prevention methods, particularly in those with extensive burns (>20%).
Tier 2	Combat lifesaver	All Tier 1 skills Tactical evacuation care	Same as Tier 1 skills
Tier 3	Combat Medic or Hospital Corpsman	All Tier 2 skills Triage Lifesaving interventions to include triage, junctional tourniquet application, use of airway adjuncts, cricothyroidotomy, oxygen administration, shock and burn resuscitation, and fracture management	Consider early surgical airway for respiratory distress or oxygen desaturation in patients with facial burns and/or at risk of inhalation injury. Burn fluid resuscitation using the Rule of Ten: initial IV/IO fluid rate is calculated as %TBSA × 10 mL/h for adults weighing 40–80 kg. For every 10 kg ABOVE 80 kg, increase initial rate by 100 mL/h. If burns are greater than 20% of TBSA, fluid resuscitation should be initiated as soon as IV/IO access is established. Resuscitation should be initiated with lactated Ringer’s, normal saline, or Hextend (if available). If Hextend is used, no more than 1000 mL should be given, followed by lactated Ringer’s or normal saline as needed. If hemorrhagic shock is also present, resuscitation for hemorrhagic shock takes precedence over resuscitation for burn shock. Administer IV/IO fluids per the TCCC guidelines Prehospital antibiotic therapy is not indicated solely for burns, but antibiotics should be given per the TCCC guidelines if indicated to prevent infection in penetrating wounds.
Tier 4	Combat paramedic or provider (including physicians, physician assistants, and Independent Duty Corpsman)	All Tier 3 skills Advanced lifesaving interventions such as endotracheal intubation and tube or finger thoracostomy	Same as Tier 3 skills

* Adapted from Butler et al. [27].

**Table 2:** Non-combat U.S. Navy surface ship and submarine causes of burn injury, 1970–2020 *.

Burn Injury Mechanism	Casualties (N)	Mortality (%) **	Most Recent Occurrence
Fire/burn/smoke inhalation (103 events)	923	13	2018
Explosion (16 events)	106	60.4	2003
Chemical exposure/inhalation injury (27 events)	104	27.9	2004
Electrocution	32	90.6	2018
Ordinance-related mishap	24	37.5	2004
Collision (14 events) ***	275	40.3	2017

* Adapted from Benham et al. [4]. ** Mortality among total casualties. *** Fires and explosions can occur during collisions depending on the circumstances.

**Table 3:** Known attacks on U.S. vessels, 1970–2020 *.

Year	Ship	War/Conflict	Mechanism	Estimated Crew	Casualties (%)	Mortality (%) **
1972	USS Goldsborough (DDG-20)	Vietnam War	Coastal artillery fire	354	5 (1.4)	3 (60)
1987	USS Stark (FFG-31)	Iran–Iraq War	Exocet missile attack	220	58 (15.6)	37 (63.8)
1988	USS Samuel B. Roberts (FFG-58)	Iran–Iraq War	Naval mine	205	10 (4.9)	0
1991	USS Princeton (CG-59)	Operation Desert Storm	Naval mine	330	3 (0.9)	0
1991	USS Tripoli (LPH-10)	Operation Desert Storm	Naval mine	2358	4 (0.17)	0
2000	USS Cole (DDG-67)	Terrorist attack	WBIED	338	54 (16)	17 (31.5)
			Totals	3805	134 (3.5%)	57 (42.5)

DDG—Destroyer; FFG—Frigate, Guided Missile; CG—Cruiser; LPH—Landing Platform Helicopter; WBIED—water-borne improved explosive device. * Adapted from Vasquez et al. and Benham et al. [3,4]. ** Mortality among total casualties.

**Table 4:** Select major fires on U.S. aircraft carriers while underway [7,55,56,57].

Year	Ship	War/Location	Crew	Location/Cause	Casualties (%)	Mortality (%) *
1954	USS Bennington (CVA 20)	Rhode Island	2600	Catapult malfunction and explosion	201 (7.7)	103 (51.2)
1966	USS Oriskany (CVA 34)	Vietnam War	3400	Forward Hangar Deck, flare	200 (5.9)	44 (22)
1967	USS Forrestal (CVA 59)	Vietnam War	5500	Flight Deck, ordinance related	295 (5.4)	134 (45.4)
1969	USS Enterprise (CVN 65)	Hawaii	5162	Flight Deck, ordinance related	343 (6.6)	28 (8.2)

* Mortality among total casualties.

**Table 5:** Casualty estimates of two hypothetical Carrier Strike Groups and one Surface Action Group *.

Ship Type	# Ships	Standard Crew Complement	Casualties per Ship	Injured Survivors	Burn Injuries in Survivors	Medical Capabilities
Aircraft Carrier (CVN)	2	5500 *Total: 11,000*	2718; 62% Fatal *Total: 5436*	1030 *Total: 2060*	309 *Total 618*	Physicians ………………………5 Nurses……………………………1 Corpsman………………………30 Ward Beds………………………52 Intensive Care Unit Beds………3 Operating Room Beds…………..1 Battle Dressing Stations………..6
Destroyer (DDG)	8	314 *Total: 2512*	136; 39% Fatal *Total: 1088*	83 *Total: 664*	25 *Total: 200*	Independent Duty Corpsman….1 Corpsman………………………..3 Ward Beds………………………..2 Battle Dressing Stations…………2
Cruiser (CG)	3	376 *Total: 1128*	162; 39% Fatal *Total: 486*	99 *Total: 297*	30 *Total: 90*	Independent Duty Corpsman….1 Corpsman………………………..3 Ward Beds……………………….2 Battle Dressing Stations………..2
Totals (%)	13	14,640	7010; 57% Fatal	3021	908 (30%)	

* Casualty estimates based on the casualties from the USS Franklin [119] and USS Stark attacks [34] and assuming a 30% burn injury rate in survivors using WWII estimates after surface kamikaze attacks [20].

## Data Availability

The data presented in this review article are openly available in reference numbers [3,5,7,21,22,23,24,25,28,29,30,31,32,36,37,38].
